# Nebivolol Desensitizes Myofilaments of a Hypertrophic Cardiomyopathy Mouse Model

**DOI:** 10.3389/fphys.2017.00558

**Published:** 2017-08-02

**Authors:** Sabrina Stücker, Nico Kresin, Lucie Carrier, Felix W. Friedrich

**Affiliations:** ^1^Department of Experimental Pharmacology and Toxicology, Cardiovascular Research Center, University Medical Center Hamburg-Eppendorf Hamburg, Germany; ^2^German Centre for Cardiovascular Research (DZHK) Hamburg, Germany

**Keywords:** nebivolol, myofilament, Ca^2+^ sensitivity, hypertrophic cardiomyopathy, *Mybpc3*, mouse, human, epigallocatechin-3-gallate

## Abstract

**Background:** Hypertrophic cardiomyopathy (HCM) patients often present with diastolic dysfunction and a normal to supranormal systolic function. To counteract this hypercontractility, guideline therapies advocate treatment with beta-adrenoceptor and Ca^2+^ channel blockers. One well established pathomechanism for the hypercontractile phenotype frequently observed in HCM patients and several HCM mouse models is an increased myofilament Ca^2+^ sensitivity. Nebivolol, a commonly used beta-adrenoceptor antagonist, has been reported to lower maximal force development and myofilament Ca^2+^ sensitivity in rabbit and human heart tissues. The aim of this study was to evaluate the effect of nebivolol in cardiac muscle strips of an established HCM *Mybpc3* mouse model. Furthermore, we investigated actions of nebivolol and epigallocatechin-gallate, which has been shown to desensitize myofilaments for Ca^2+^ in mouse and human HCM models, in cardiac strips of HCM patients with a mutation in the most frequently mutated HCM gene *MYBPC3*.

**Methods and Results:** Nebivolol effects were tested on contractile parameters and force-Ca^2+^ relationship of skinned ventricular muscle strips isolated from *Mybpc3*-targeted knock-in (KI), wild-type (WT) mice and cardiac strips of three HCM patients with *MYBPC3* mutations. At baseline, KI strips showed no difference in maximal force development compared to WT mouse heart strips. Neither 1 nor 10 μM nebivolol had an effect on maximal force development in both genotypes. 10 μM nebivolol induced myofilament Ca^2+^ desensitization in WT strips and to a greater extent in KI strips. Neither 1 nor 10 μM nebivolol had an effect on Ca^2+^ sensitivity in cardiac muscle strips of three HCM patients with *MYBPC3* mutations, whereas epigallocatechin-gallate induced a right shift in the force-Ca^2+^ curve.

**Conclusion:** Nebivolol induced a myofilament Ca^2+^ desensitization in both WT and KI strips, which was more pronounced in KI muscle strips. In human cardiac muscle strips of three HCM patients nebivolol had no effect on myofilament Ca^2+^ sensitivity.

## Introduction

Hypertrophic cardiomyopathy (HCM) is the most frequent cardiac genetic disease primarily caused by mutations in sarcomeric protein genes (Friedrich and Carrier, 2012; Maron et al., 2014; Ho et al., 2015). The most commonly mutated genes are *MYBPC3* (encoding cardiac myosin-binding protein C) and *MYH7* (encoding β-myosin-heavy chain) (Walsh et al., 2017). HCM is principally characterized by asymmetric left ventricular hypertrophy, diastolic dysfunction and myocardial disarray (Elliott et al., 2008). Current pharmacological treatment of HCM mainly relies on beta-adrenoceptor (AR) and Ca^2+^ channel blockers, which improve clinical symptoms, partially prevent arrhythmias and improve diastolic dysfunction by prolonging left ventricular (LV) filling time and reducing outflow tract obstruction (Maron et al., 2003; Gersh et al., 2011; Spoladore et al., 2012; Hamada et al., 2014; Tardiff et al., 2015). Increased Ca^2+^ sensitivity seems to be a common factor in HCM as seen in animal HCM models (Tardiff et al., 1999; Cazorla et al., 2006; Pohlmann et al., 2007; Vignier et al., 2009; Fraysse et al., 2012; Barefield et al., 2014; Wijnker et al., 2016), and human HCM samples (Jacques et al., 2008; van Dijk et al., 2009, 2012). The increased Ca^2+^ response may contribute to diastolic dysfunction and arrhythmias (Morimoto et al., 1998; Baudenbacher et al., 2008). Even though the mechanisms accountable for increased myofilament Ca^2+^ sensitivity remain unclear, targeting this pathomechanism by interventions decreasing myofilament Ca^2+^ sensitivity may be an attractive alternative for the treatment of HCM and improvement in symptoms (Jagatheesan et al., 2007; Alves et al., 2014; Tardiff et al., 2015). Among beta-AR blockers that are commonly used in the treatment of cardiovascular diseases, nebivolol has been reported to lower maximal force development and to desensitize rabbit and human cardiac myofilaments to Ca^2+^ (Zeitz et al., 2000; Janssen et al., 2001). However, the effects of nebivolol were never evaluated in HCM models with increased myofilament Ca^2+^ sensitivity. An established HCM mouse model carrying the human c.772G>A *MYBPC3* mutation is the *Mybpc3* KI mouse model (Vignier et al., 2009). This mutation was frequently found in unrelated HCM patients in Tuscany and is associated with a bad prognosis (Richard et al., 2003; Girolami et al., 2006; Ho et al., 2015). At the homozygous state, this mouse model exhibits HCM-like features such as left ventricular hypertrophy, diastolic dysfunction and increased myofilament Ca^2+^ sensitivity (Vignier et al., 2009; Fraysse et al., 2012). We recently showed that epigallocatechin-3-gallate (EGCg), a major component of green tea, hastened relaxation and Ca^2+^ transient in KI cardiomyocytes and decreased Ca^2+^ sensitivity of KI myofilaments (Friedrich et al., 2016). In this study, we investigated nebivolol effects on myofilament Ca^2+^ sensitivity in *Mybpc3* KI cardiac muscle strips. We furthermore assessed nebivolol and EGCg effects in cardiac strips of three HCM patients with *MYBPC3* mutations.

## Materials and methods

### Human samples

Human myocardial samples were obtained from three HCM patients carrying heterozygous *MYBPC3* mutations (c.1960C>T, c.2308G>A, c.2234A>G) who underwent septal myectomy due to outflow tract obstruction. The material was taken with written informed consent of the donor and with written approval of the local ethical boards. The study has been carried out in accordance with The Code of Ethics of the World Medical Association (Declaration of Helsinki).

### Animals

The *Mybpc3* KI cardiomyopathy mouse model was generated by the targeted insertion of a G>A transition on the last nucleotide of exon 6 (Vignier et al., 2009; Fraysse et al., 2012; Schlossarek et al., 2012, 2014; Gedicke-Hornung et al., 2013; Mearini et al., 2013, 2014; Stohr et al., 2013; Friedrich et al., 2014; Najafi et al., 2015; Thottakara et al., 2015; Flenner et al., 2016, 2017). Mice were maintained on the C57 background. As controls, *Mybpc3* WT mice of the same background were used. The study was exerted in accordance with the recommendations of the guide for the care and use of laboratory animals published by the NIH (Publication No. 85–23, revised 2011 published by National Research Council) and comply with the ARRIVE guidelines (http://www.nc3rs.org.uk/arrive-animal-research-reporting-vivo-experiments). All experimental procedures were in accordance with the German Law for the Protection of Animals and the protocol was approved by the Ministry of Science and Public Health of the City State of Hamburg, Germany (Org 653).

### Skinned ventricular trabeculae force measurements

For the determination of force-Ca^2+^ relationships, trabeculae were prepared from ventricular endocardial surface of WT and KI mice or human myocardium of a septal myectomy (Flenner et al., 2016; Friedrich et al., 2016). Dimensions of strips were 2.91 ± 0.14 mm in length, 0.36 ± 0.01 mm in width and 0.11 ± 0.01 mm^2^ in cross-sectional area (CSA), calculated by 2πr^2^ assuming a circular shape, n_WT_ = 17, n_KI_ = 18, n_human_ = 57. Strips were permeabilized in relaxing solution (pCa 9) in EGTA-buffer (5.89 mM Na_2_ATP, 14.5 mM CrP, 6.48 mM MgCl_2_, 40.76 mM Kprop, 100 mM BES and 7 mM EGTA, pH 7.1) (Kooij et al., 2010; Stoehr et al., 2014) containing 1% Triton X-100 at 4°C for 18 h. The next day strips were either directly used for measurements or stored at −20°C in a 50% glycerol/relaxing solution containing protease inhibitors (EDTA-free, complete tablets, mini, Roche). The Ca^2+^-sensitivity of permeabilized cardiac strips was evaluated using a fiber test system (1400A; Aurora Scientific) by mounting them between a force transducer and a length controller. Strips were stretched above slack length until they developed force in activating solution (pCa 4.5) at 15°C. For contraction-relaxation cycles strips were kept in pCa 9 to achieve full relaxation. Then they were moved to pCa 4.5 until maximal force development was reached. Maximal force was related to cross-sectional area (mN/mm^2^). For force-Ca^2+^-curves they were exposed to increasing Ca^2+^ concentrations from pCa 9 to pCa 4.5 in EGTA-buffer. Force development was measured in each pCa solution. Measurements were repeated in the presence of 1 or 10 μM nebivolol (nebivolol hydrochloride, Sigma Life Sciences) or 30 μM epigallocatechin-gallate (Sigma Life Sciences) after 5 min preincubation in relaxing solution (Flenner et al., 2016; Friedrich et al., 2016). In every second measurement, nebivolol was tested first and a control measurement was performed 5 min after nebivolol washout to exclude time-dependent force rundown. Each strip was measured in a pairwise manner (paired analysis baseline vs. intervention) serving as its own control. Data were analyzed using the Hill equation (Hill et al., 1980), with pCa_50_ as the free Ca^2+^ concentration which yields 50% of the maximal force and nH representing the Hill coefficient. The pCa_50_ represents the measure of myofilament Ca^2+^ sensitivity.

### Statistical analysis

Data were expressed as mean ± SEM. Comparisons were performed by paired or unpaired Student's *t*-test and with one-way ANOVA, followed by Bonferroni's post-test as indicated in the figure legends. Concentration response curves were fitted to the data points and force-pCa relationship comparison was done by using extra sum-of-squares *F*-test (GraphPad, Prism 6). A value of *P* < 0.05 was considered statistically significant.

## Results

### Nebivolol (1 and 10 μM) has no effect on maximal force development in permeabilized cardiac strips of *Mybpc3* WT and KI mice

**Figure 1:**
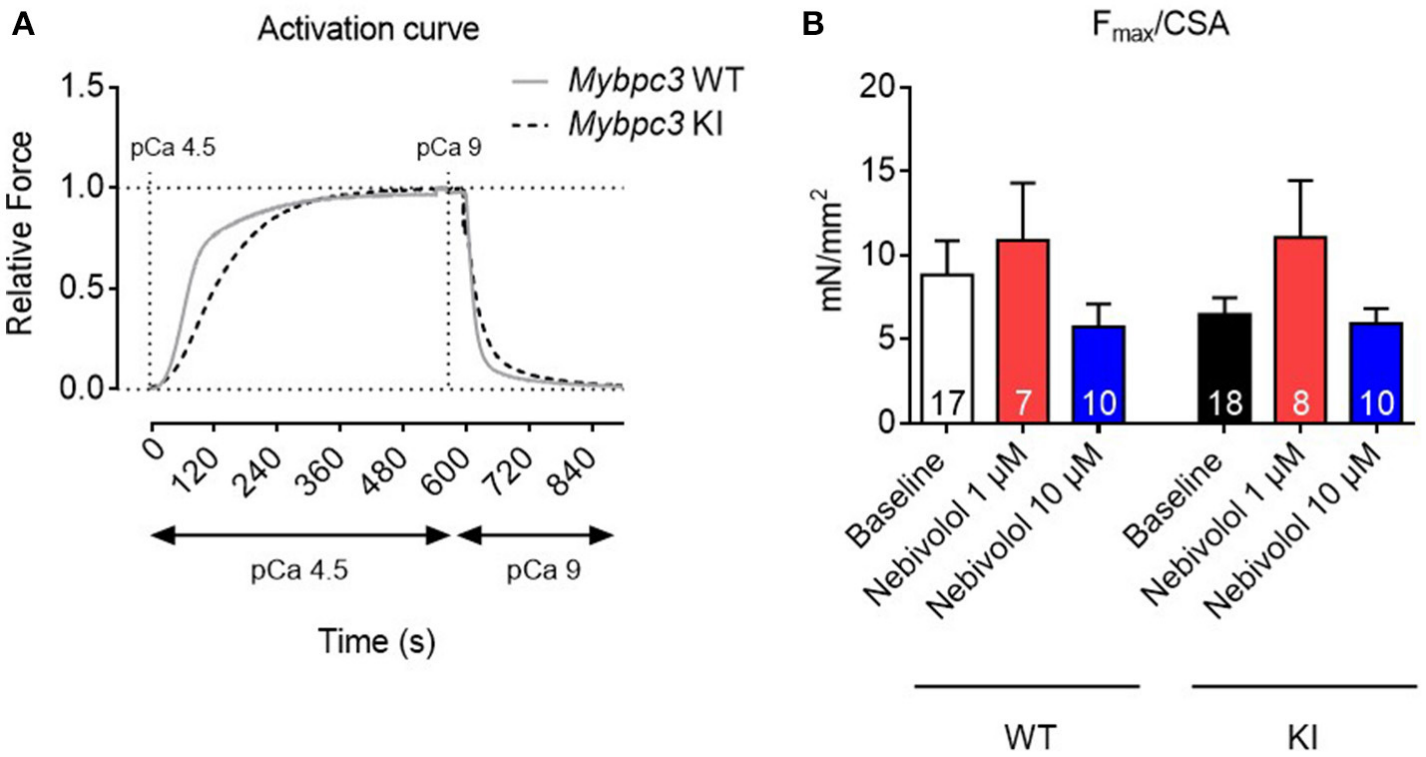
Contractile parameters of permeabilized cardiac muscle strips of *Mybpc3* WT and KI mice with and without nebivolol treatment. **(A)** Representative normalized activation curves of *Mybpc3* WT (gray) and KI (black striped) mouse strips. **(B)** Quantification of maximal force development related to cross sectional area (CSA) in pCa 4.5 ± nebivolol 1 and 10 μM; number of strips is indicated in the bars.

The hypercontractile phenotype observed in HCM patients could be attributed to an increased myofilament Ca^2+^ sensitivity. Since nebivolol has been reported to lower maximal force development and to desensitize rabbit and human cardiac myofilaments (Zeitz et al., 2000; Janssen et al., 2001), we investigated nebivolol effects on myofilament Ca^2+^ sensitivity in cardiac muscle strips of *Mybpc3* KI mice with an increased myofilament Ca^2+^ sensitivity (Vignier et al., 2009; Fraysse et al., 2012; Flenner et al., 2016; Friedrich et al., 2016). There are conflicting reports concerning the effects of nebivolol on maximal force development and myofilament Ca^2+^ sensitivity in cardiac muscle strips. Whereas Zeitz et al. reported that 1 μM nebivolol lowered maximal force development and myofilament Ca^2+^ sensitivity in skinned trabeculae (Zeitz et al., 2000), Bundkirchen and colleagues did not observe such an effect at 10 μM (Bundkirchen et al., 2001). We therefore used 1 and 10 μM for our experiments. To investigate nebivolol effects on force development we measured contraction-relaxation cycles in skinned myofilaments (Figure 1A). Analysis showed that maximal force development in baseline conditions did not significantly differ between WT and KI muscle strips (Figure 1B). Neither 1 nor 10 μM nebivolol had an effect on maximal force development (Figure 1B).

### Nebivolol decreases myofilament Ca^2+^ sensitivity to a greater extent in KI than in WT skinned ventricular trabeculae

**Figure 2:**
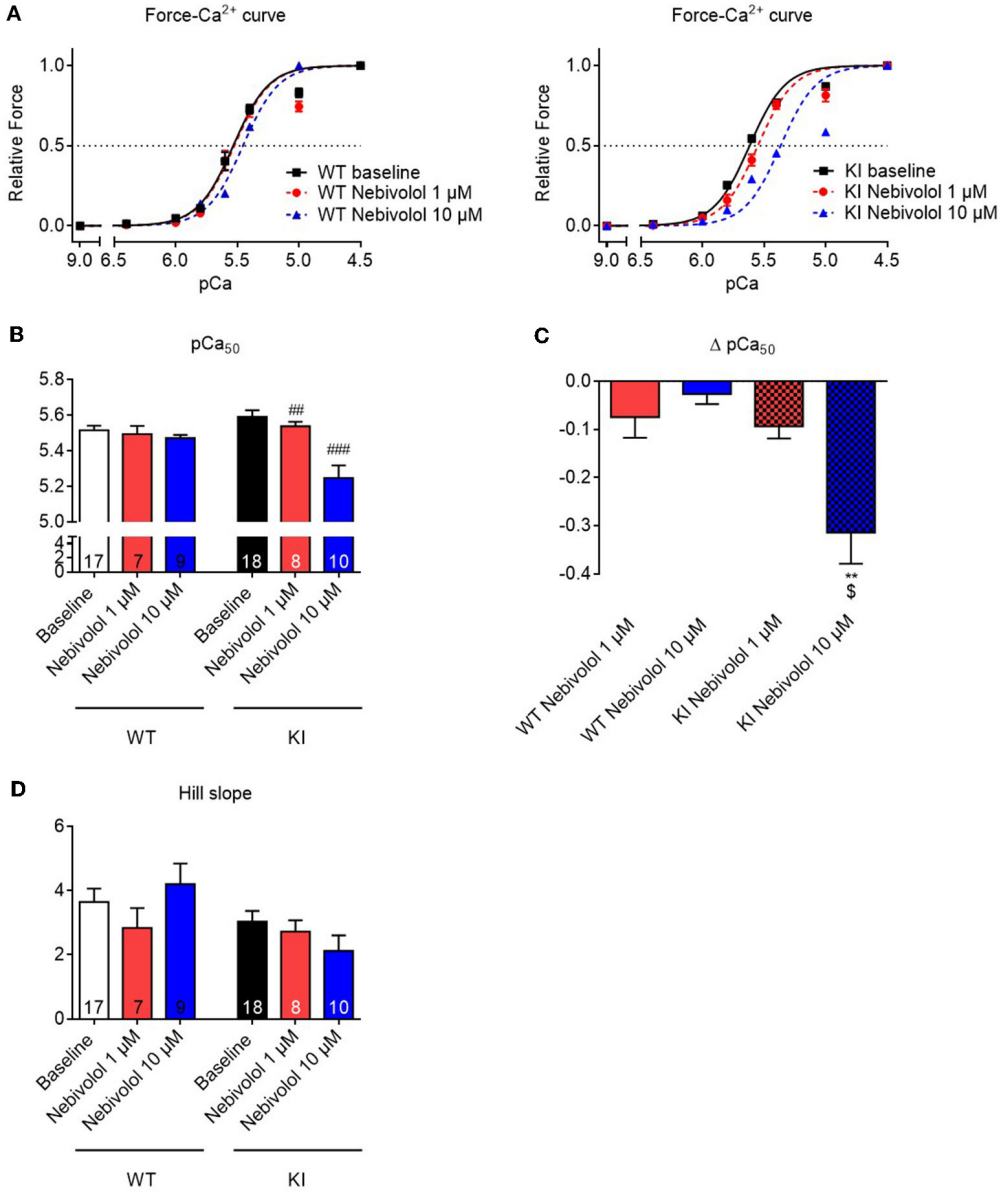
Force-Ca^2+^ relationship of permeabilized cardiac muscle strips of *Mybpc3* WT and KI mice with and without nebivolol treatment. **(A)** Force-Ca^2+^ relationship in WT (left) and KI (right) strips ±nebivolol 1 and 10 μM. **(B)** pCa_50_ representing the negative logarithm of the calcium concentration needed for half-maximal activation ±nebivolol 1 and 10 μM. **(C)** Delta of pCa_50_ ± nebivolol 1 and 10 μM. **(D)** nHill coefficient (Hill slope) ± nebivolol 1 and 10 μM. **P < 0.01 vs. WT in the same condition and ^$^*p* < 0.05 vs. KI 1 μM, unpaired Student's *t*-test; ^##^*P* < 0.01 and ^###^*P* < 0.001 vs. baseline, paired Student's *t*-test, concentration response curves were fitted to the data points and curve comparison was done by using extra sum-of-squares *F*-test; number of strips is indicated in the bars.

Nebivolol has been reported to decrease Ca^2+^ sensitivity in rabbit and human cardiac myofilaments (Zeitz et al., 2000; Janssen et al., 2001). To assess whether this is also the case in myofilaments of *Mybpc3* KI mouse hearts, we measured force-pCa relationships in skinned ventricular trabeculae from WT and KI mice. In analogy to the experiments on maximal force development, we performed force-pCa relationships in the absence and presence of 1 and 10 μM nebivolol, respectively. As observed before (Fraysse et al., 2012; Flenner et al., 2016; Friedrich et al., 2016), skinned KI trabeculae showed a higher pCa_50_ than WT trabeculae in baseline conditions, representing higher myofilament Ca^2+^ sensitivity (Figures 2A,B). In WT strips, only incubation with 10 μM (by extra sum-of-squares *F*-test) shifted the force-Ca^2+^ relationship to the right resulting in a lower pCa_50_, whereas in KI strips both 1 and 10 μM lowered pCa_50_ (Figures 2A,B) indicating myofilament Ca^2+^ desensitization. This effect was concentration-dependent since incubation with 10 μM nebivolol induced a stronger shift (Δ pCa_50_) to the right (Figure 2C). The nHill coefficient (Hill slope) as a an index for myofilament co-operativity did not differ between the genotypes neither with nor without nebivolol (Figure 2D).

### Nebivolol does not impact on maximal force development or myofilament Ca^2+^ sensitivity in muscle strips derived from cardiac tissue of HCM patients with *MYBPC3* mutations

**Figure 3:**
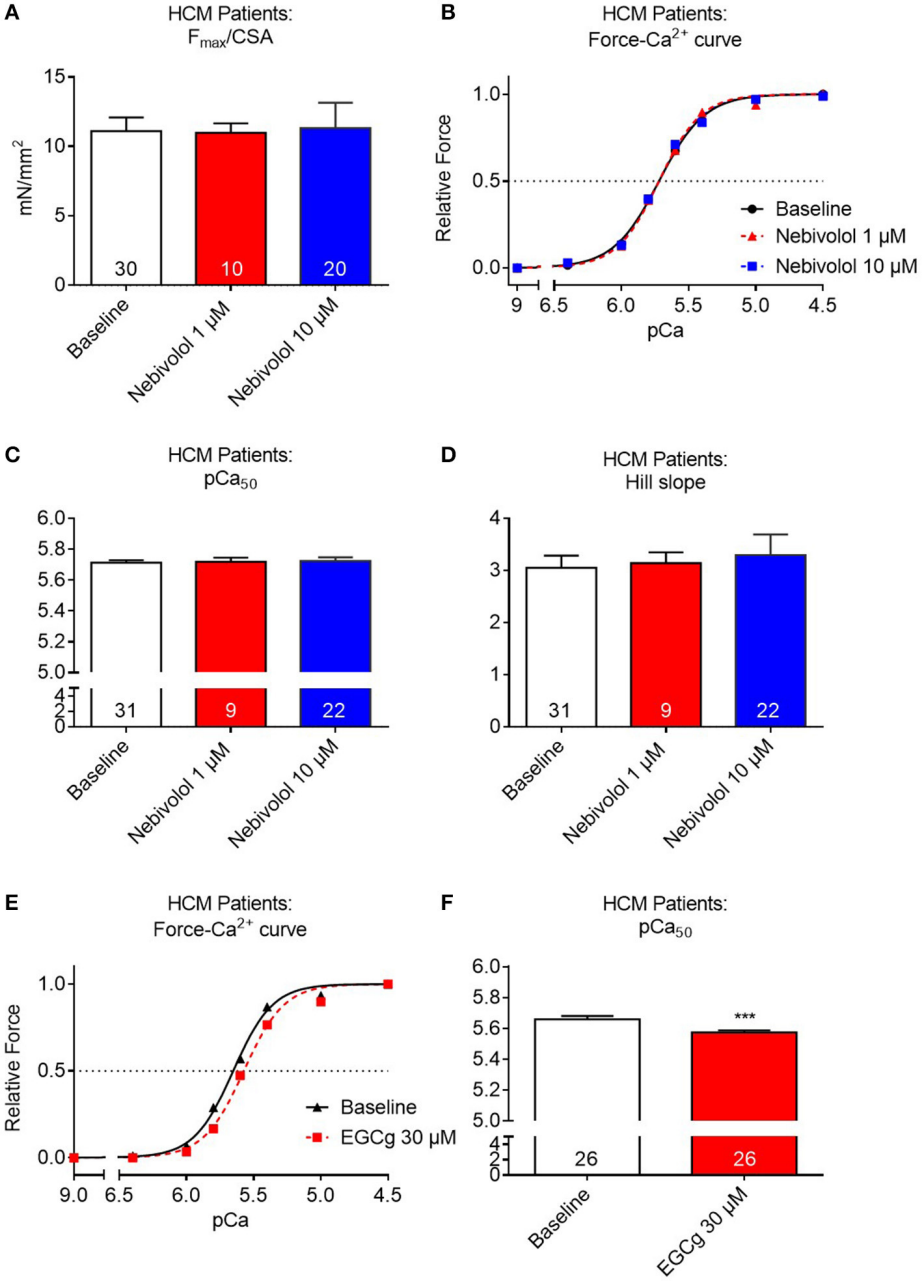
Contractile parameters of permeabilized cardiac muscle strips of three human HCM patients carrying different *Mybpc3* mutations in the absence or presence of nebivolol. **(A)** Quantification of maximal force development related to cross sectional area (CSA) in pCa 4.5 ±nebivolol 1 and 10 μM. **(B)** Force-Ca^2+^ relationship ±nebivolol 1 and 10 μM. **(C)** pCa_50_ representing the negative logarithm of the calcium concentration needed for half-maximal activation ±nebivolol 1 and 10 μM. **(D)** nHill coefficient (Hill slope) ±nebivolol 1 and 10 μM. **(E)** Force-Ca^2+^ relationship ±EGCg 30 μM. **(F)** pCa_50_ ±EGCg 30 μM. ***P < 0.001 vs. baseline, paired Student's *t*-test. Concentration response curves were fitted to the data points and curve comparison was done by using extra sum-of-squares *F*-test; number of strips is indicated in the bars.

Since nebivolol has been reported to desensitize human myofilaments for Ca^2+^ and since both 1 and 10 μM nebivolol had induced a right-ward shift of the force-pCa curves in *Mybpc3* KI cardiac muscle strips we sought to investigate whether it would also affect myofilament Ca^2+^ sensitivity in human HCM tissue. Similar to the experiments performed with mouse cardiac strips we investigated the effects of 1 and 10 μM nebivolol on contraction-relaxation cycles in muscle strips of three HCM patients carrying different *MYBPC3* mutations. Neither 1 nor 10 μM nebivolol had an influence on F_max_ (Figure 3A). Furthermore, no shift in Ca^2+^ sensitivity nor change in Hill slope was observed for the Ca^2+^-dependent force development (Figures 3B–D). In contrast and as reported before by us in *Mybpc3* WT and KI strips (Friedrich et al., 2016), incubation with a positive control compound (30 μM epigallocatechin-gallate; EGCg) shifted the force-Ca^2+^-relationship to the right in strips from the same three HCM patients (Figures 3E,F).

## Discussion

HCM patients often present with a normal to supranormal systolic function and diastolic dysfunction. To counteract this hypercontractility, guideline therapies advocate treatment with beta-AR and Ca^2+^ channel blockers. One well established pathomechanism for the hypercontractile phenotype frequently observed in HCM patients and several HCM mouse models is an increased myofilament Ca^2+^ sensitivity (Morimoto et al., 1998; Robinson et al., 2007; Huke and Knollmann, 2010; Kimura, 2010; Fraysse et al., 2012; Moore et al., 2012; van Dijk et al., 2012; Barefield et al., 2014; Elliott et al., 2014; Flenner et al., 2016; Friedrich et al., 2016). Nebivolol, a commonly used beta-AR antagonist, has been reported to lower maximal force development and myofilament Ca^2+^ sensitivity in rabbit and human heart tissues (Zeitz et al., 2000; Janssen et al., 2001). Given the hypercontractile phenotype mentioned above, these pleiotropic actions predestine it for HCM treatment. The aim of this study was to evaluate whether nebivolol would exert similar effects in permeabilized myofilaments of an *Mybpc3* HCM mouse model and of HCM patients with mutations in the most frequently mutated gene *MYBPC3*. The main findings of this study were: (1) At baseline, permeabilized left ventricular trabeculae isolated from *Mybpc3* KI mouse hearts showed no difference in maximal force development compared to WT mouse heart strips. (2) Neither 1 nor 10 μM nebivolol had an effect on maximal force development in both genotypes. (3) 10 μM nebivolol induced myofilament Ca^2+^ desensitization in both WT and KI strips and this effect was more pronounced in KI muscle strips, respectively. (4) Nebivolol had no effect on Ca^2+^ sensitivity in cardiac muscle strips of three HCM patients with *MYBPC3* mutations, whereas 30 μM of EGCg induced a right shift in the force-Ca^2+^ curve.

In mice, nebivolol did not influence maximal force development. On the other hand, it affected myofilament Ca^2+^ sensitivity in mouse strips. The mechanism behind this is unknown so far. In analogy to the mouse results, 1 and 10 μM nebivolol had no effect on maximal force development in human tissues. In contrast to the observations made in mouse strips, it did not impact on myofilament Ca^2+^ sensitivity.

The reason why nebivolol exerted a myofilament Ca^2+^ desensitizing effect in KI strips at both 1 and 10 μM, whereas in WT strips only 10 μM had an effect and no effect at all in human HCM tissues remains unclear. As mentioned before, discrepancies concerning the effects of nebivolol on maximal force development and myofilament Ca^2+^ sensitivity in cardiac muscle strips have been previously described. Whereas Zeitz et al. reported that 1 μM nebivolol lowered maximal force development and myofilament Ca^2+^ sensitivity in skinned rabbit and human trabeculae (Zeitz et al., 2000), Bundkirchen and colleagues did not observe such an effect at 10 μM in human tissue (Bundkirchen et al., 2001). The findings are contradictory but could be explained by either differences (i) between species (rabbit vs. mouse vs. human), (ii) in experimental setups, (iii) the functional status of the tissues (non-failing vs. failing), or (iv) a combination of it. Interspecies- or setup-dependent differences have been reported in permeabilized strip experiments with other drug interventions (Lues et al., 1988; Edes et al., 1995). Zeitz et al. used non-failing cardiac tissue from rabbit and explanted human tissue from end-stage failing myocardium from patients undergoing heart transplantation and saw an effect in tissues of both species. In another study the same group did not observe any effect on maximal force development in human explanted heart muscle preparations (Janssen et al., 2001). We observed no effect on maximal force development in neither mouse nor human tissue, but on myofilament Ca^2+^ sensitivity in mouse tissue, which was more pronounced in the KI strips. Similar to this study we observed in a previous study with skinned trabeculae that EGCg, another compound with Ca^2+^-desensitizing properties had a more profound effect on strips of the KI than the WT genotype (Friedrich et al., 2016). This is also compatible with results of a study in which the Ca^2+^-desensitizing effect of ranolazine was only present in KI, but not in WT muscle strips (Flenner et al., 2016). The reason for the difference between KI and WT is unclear but could be related to the higher baseline myofilament Ca^2+^ sensitivity in KI or to the proposed antioxidative activity of ranolazine, which might be important in a potentially hyperoxidized KI tissues (Lovelock et al., 2012; Flenner et al., 2016). In analogy to the study of Bundkirchen et al., in which nebivolol had no effect in explanted left ventricular tissue of patients with dilated cardiomyopathy, we did not observe any effect on Ca^2+^ sensitivity at 1 or 10 μM in the HCM samples. In contrast, 30 μM of the positive control compound EGCg, which has been suggested to alter the interaction between cardiac troponin C and I and therefore the sensitivity of the myofilaments to Ca^2+^ (Liou et al., 2008; Robertson et al., 2009), induced a rightward shift in the force-Ca^2+^ curve in human HCM strips. EGCg has been shown to lower the myofilament Ca^2+^ sensitivity in a transgenic HCM mouse model expressing a human cardiac troponin T mutant (Tadano et al., 2010) and in HCM-associated human cardiac troponin I and T mutants (Tadano et al., 2010; Warren et al., 2015; Messer et al., 2016). Similarly, we reported that 30 μM EGCg decreased Ca^2+^ sensitivity in our *Mybpc3* KI mouse model that carries a frequent *Mybpc3* HCM mutation (Friedrich et al., 2016). EGCg action on myofilament Ca^2+^ sensitivity in cardiac muscle strips of patients carrying a heterozygous *MYBPC3* mutation indicates that the human strips can be desensitized for Ca^2+^.

Yet the precise mechanism of Ca^2+^-desensitization of nebivolol in mouse heart tissue remains unaddressed. Nebivolol is a third-generation beta-AR antagonist that exhibits vasodilating properties, most likely due to stimulation of nitric oxide synthase (Cockcroft et al., 1995). Since it was shown to attenuate hydroxyl radical-induced myocardial damage which has been associated with altered intracellular calcium handling and calcium overload of the myocytes (Josephson et al., 1991; Janssen et al., 1999; Piccini et al., 2012), it was proposed that nebivolol has direct free-radical scavenging properties (Janssen et al., 1999). Whether such an indirect effect is the main reason for a decrease in myofilament Ca^2+^ sensitivity or another direct mechanism on the moyfilaments, such as binding to the C-terminal region of cardiac troponin C altering the interaction between cTnC and cTnI as in the case of the positive control compound EGCg (Liou et al., 2008; Robertson et al., 2009), exists, remains to be shown.

Clinically, beta-AR-antagonists are the mainstay of HCM therapy (Elliott et al., 2014). They are thought to potentially improve diastolic filling by a negative chronotropic effect. Some studies support the use of beta-AR-antagonists in HF patients with preserved ejection fraction (EF) but impaired relaxation similar to diastolic dysfunction seen in HCM patients (Lund et al., 2014). Recent data suggest that the effect of nebivolol is similar in HF patients with reduced and preserved EF (van Veldhuisen et al., 2009). This initiated the design of a still ongoing trial (https://clinicaltrials.gov/ct2/show/NCT02619526) investigating the effects of nebivolol and carvedilol on diastolic function of the left ventricle in older HF patients with preserved EF (Park and Park, 2016).

## Limitations of the study

(1) The *Mybpc3* KI model shows many HCM characteristics only at the homozygous state. Moreover, *Mybpc3* KI mice present a reduced EF. These two points are in contrast to the more common findings in HCM patients who present left ventricular hypertrophy, interstitial fibrosis and diastolic dysfunction with heterozygous mutation states and normal or even supra-normal EF. (2) Our study does not explain the precise mechanism of Ca^2+^-desensitization of nebivolol in mouse heart tissue. (3) The Ca^2+^ desensitizing effect of nebivolol in mouse tissues occurred at concentrations which were above the plasma concentrations (0.8–3.7 nM) observed in humans (Stoschitzky et al., 2004; Prisant, 2008). (4) Even though we did not observe any nebivolol effect in the human strips in this study, this observation cannot be generalized to all HCM patients since the number of tissues was low and they were derived from HCM patients carrying only mutations in *MYBPC3*.

## Conclusion

Nebivolol had no effect on maximal force, but induced a myofilament Ca^2+^ desensitization in both WT and KI mouse cardiac muscle strips, which was more pronounced in KI muscle strips. In human cardiac muscle strips, nebivolol had no effect on force development and myofilament Ca^2+^ sensitivity. Further studies should investigate the exact target and mechanism for Ca^2+^ desensitization in mouse cardiac tissues in order to be able to develop modified compounds with even more potency and specificity for use in human tissue.

## Author contributions

SS and NK: Isolation and treatment of cardiac muscle strips and execution of experiments. LC: Analysis and interpretation of data, and correction of the manuscript. FF: Conception and design of research, execution of experiments, analysis and interpretation of data, figure preparation, and drafting of the manuscript. All authors critically discussed the results, and reviewed and approved the manuscript before submission.

### Conflict of interest statement

The authors declare that the research was conducted in the absence of any commercial or financial relationships that could be construed as a potential conflict of interest.
